# Increased systemic zonula occludens 1 associated with inflammation and independent biomarker in patients with hepatocellular carcinoma

**DOI:** 10.1186/s12885-018-4484-5

**Published:** 2018-05-18

**Authors:** Amit Kumar Ram, Biju Pottakat, Balasubramaniyan Vairappan

**Affiliations:** 1 Liver Diseases Research Lab, Department of Biochemistry, Jawaharlal Institute of Postgraduate Medical Education and Research (JIPMER), Dhanvantari Nagar, Puducherry, 605006 India; 2 Department of Surgical Gastroenterology, Jawaharlal Institute of Postgraduate Medical Education and Research (JIPMER), Pondicherry, 605006 India

**Keywords:** Tight junction, Liver cancer, Inflammation, Blood biomarker, Hepatic marker enzymes

## Abstract

**Background:**

Hepatocellular carcinoma (HCC) is a frequent type of primary liver cancer, and its prevalence is increasing worldwide. Indeed, the underlying molecular mechanism is not well understood. Previous studies have shown evidence that tight junction (TJ) components were correlated with carcinogenesis and tumor development. Our aims were to determine the serum levels of tight junction protein Zonula Occludens (ZO)-1 and an inflammatory marker such as high-sensitive C-reactive protein (hs-CRP) in HCC patients compared to healthy volunteers and also to identify the association between ZO-1 and inflammation in HCC.

**Methods:**

Thirty HCC patients and 30 healthy volunteers were recruited in the current study. Clinical data regarding child class, BCLC staging, the number of lesions, tumor size, absence or presence of metastasis, cirrhosis and hepatitis infection were also collected in HCC patients. Plasma ZO-1 and serum hsCRP were analyzed by EIA and ELISA respectively and biochemical parameters by autoanalyser (AU680 Beckman Coulter, USA). Furthermore, hepatic ZO-1 protein expression and tissue localization were examined.

**Results:**

Compared to healthy individuals, the serum levels of bilirubin, alanine aminotransferase (ALT), aspartate aminotransferase (AST), gamma-glutamyltransferase (GGT) and alkaline phosphatase (ALP) were elevated significantly (*P* < 0.0001) whilst serum albumin level was significantly (P < 0.0001) decreased in HCC patients. Furthermore, tight junction protein ZO-1 concentration was significantly elevated in HCC patients compared to control subjects (648 ± 183.8 vs. 396.4 ± 135.8 pg/ml, respectively; *P* < 0.0001). Serum hsCRP level was also significantly increased in HCC patients compared to control subjects (17.25 ± 3.57 vs. 5.54 ± 2.62 mg/L, respectively; *P* < 0.0001). Moreover, decreased protein expression of ZO-1 was found in liver tissue obtained from HCC patients.

**Conclusion:**

Our findings show for the first time that the systemic concentration of ZO-1 was significantly elevated in HCC patients and is positively correlated with inflammatory markers. Thus, the current study showing evidence that inflammation promotes plasma ZO-1 concentration and raises the possibility that it could be used as a potential diagnostic biomarker for HCC progression.

**Electronic supplementary material:**

The online version of this article (10.1186/s12885-018-4484-5) contains supplementary material, which is available to authorized users.

## Background

Hepatocellular carcinoma (HCC) is the fifth most common primary liver cancer causing more than half a million deaths annually worldwide [1]. HCC progression is alarming in Asia, where hepatitis B virus infection is endemic [2, 3]. It is a complex disease with a poor prognosis, whose pathogenesis is poorly understood. HCC occurrence and mortality rates are increasing in many regions around the globe, specifying a need for the better cure. The most common risk factor for HCC is cirrhosis because of chronic heavy alcohol consumption [4], chronic hepatitis B and C virus infections [1, 3, 5]. Furthermore, inflammation also a key player involved in HCC development [6]. Diabetes [7], cigarette smoking [8] and long-term use of oral contraceptives also appear to be independent risk factors for HCC [8].

The contribution of inflammation to carcinogenesis has received major attention in hepatocarcinogenesis [9, 10]. Epidemiological evidence also suggests that up to 25% of all cancers are due to chronic low-grade inflammation or infection [10, 11]. Most HCC develops in the cirrhotic liver after prolonged inflammation, supporting the hypothesis that inflammation contributes to cancer development [12]. In this context, many published literature has shown evidence that alcohol abuse and hepatitis viral infections lead to chronic inflammation of the liver and are responsible for the progression of HCC worldwide [1, 3, 10, 11]. Moreover, 90% of HCC patients have a natural history of unresolved inflammation [11]. Hence, such considerations are extremely relevant in the design of new preventive approaches to the reduction of cancer risk. Furthermore, the treatment options for HCC have improved indeed; precise diagnostic or prognostic biomarkers are still lacking for the management of HCC.

Tight junctions (TJ) are not only a barrier but also function as a fence to restrict molecules across different cell types of the body based on the charge and size [13]. Furthermore, TJ of hepatocytes play an essential role in the blood-biliary barrier [14]. Zonula occludens 1 (ZO1) is a first tight junction phosphoprotein identified with the molecular weight of 210–225 kDa. ZO-1 appeared in the submembranous domain of tight junction in epithelia and endothelia [15, 16], binds to actin and the integral tight junction proteins occludin and claudins and junctional adhesion molecule (JAM) [16]. Diminished ZO-1 expression was found in many cancers and may closely associate with patient prognosis [17–19]. Indeed, the blood concentration of ZO-1 and its correlation with inflammation in HCC remains unknown. The aim of the current study was to analyze blood ZO-1 concentration and its association with an inflammatory marker such as hsCRP in HCC cases and control subjects.

## Methods

This study was a case control study, conducted in the Departments of Biochemistry and Surgical Gastroenterology, Jawaharlal Institute of Post Graduate Medical Education and Research (JIPMER) from March 2014 to March 2016 after obtaining approval from the Institute Ethics Committee (Human Studies) based on Ethical Guidelines of the Helsinki Declaration of 1975, as revised in 1983. Before recruitment, all the study participants were given written informed consent concerning the background and procedure of this study.

### Study participants

We had recruited both male and female aged 18–75 years, who were admitted in the Department of Surgical Gastroenterology, JIPMER. They were diagnosed as HCC (*n* = 30) based on the histological, radiological findings by Ultrasonography (USG), Magnetic Resonance Imaging (MRI), Computed Tomography (CT), or raised α-fetoprotein (AFP) levels. The majority of the HCC patients were verified by tumor biopsy or USG guided Fine Needle Aspiration Cytology (FNAC) as per European Association for the Study of the Liver (EASL) diagnostic criteria. Tissue blocks were taken from surgically removed liver tumors of HCC patients and surrounding nontumorous liver tissues of HCC patients (histologically proven control). Histological and cytological diagnoses of HCC had confirmed by the pathologist, Department of Pathology, JIPMER. Radiological tumor characteristics (Number of Lesions, Tumor size, extrahepatic metastasis) had derived from diagnostic CT or MRI scan which was evaluated by the Radiologist. Disease severity was assessed by Child-Pugh score, and HCC severity was staged according to the Barcelona Clinic Liver Cancer (BCLC) staging system. Age and gender matched healthy volunteer who were physically and mentally normal with normal liver function tests and without the history of recent infections or any tumor were included as controls (*n* = 30).

### Sample collection

5 ml of blood sample was collected from all the HCC patients and the control subjects in heparinized or EDTA coated tube. Blood was spun at 3500 rpm for 10 min at standard room temperature. Serum or plasma was separated and routine biochemistry tests were done immediately and the remaining samples (serum or plasma) were stored at − 80 °C deep freezer for ZO-1 and hsCRP measurements.

### Immunohistochemistry

HCC liver and surrounding non-tumorous liver tissues of HCC patients were fixed in 10% buffered formalin and embedded in paraffin. Three to five micrometer thick sections were stained with hematoxylin for 10 min and with eosin for 1 min to establish the diagnosis and select areas for immunohistochemistry. Silane coated tissue slides were used for immunohistochemistry. ZO-1 was detected by immunohistochemistry using rabbit polyclonal antibody against ZO-1 (ThermoFisher Scientific, USA). Deparafinized sections were blocked for endogenous peroxidase activity with 10% H_2_O_2_ in phosphate buffer for 10 min. Antigen retrieval was performed using citrate buffer in Decloaking system at 110 °C for 10 min. Primary antibodies were used in dilution of 1:100 and incubated at room temperature for 1Hr. To exclude nonspecific binding, negative controls were incubated with secondary antibody only. Immunostaining was examined in noncancerous and HCC liver tissues using Evos FLc cell imaging system (Life technologies, USA).

### Analysis of clinical parameters

Biochemical parameters such as alanine aminotransferase (ALT), aspartate aminotransferase (AST), alkaline phosphatase (ALP), gamma-glutamyltransferase (GGT), albumin, total protein and bilirubin concentrations were measured by AU680 Beckman Coulter autoanalyser, USA. Serum AFP level was measured by Chemiluminicsence using ADVIA Centaur CP immunoassay system (Siemens) at the time of diagnosis.

### Measurement of high-sensitive C reactive protein

Inflammatory marker such as hs-CRP concentration was analysed by ELISA using a commercially available kit from CALBIOTECH, USA.

### Measurement of zonula occludens 1

Plasma ZO-1 concentration was analysed by ELISA using a commercially available kit from CUSABIO USA.

### Western blot analysis

Freshly collected liver tissue from HCC and noncancerous control was snap frozen immediately in liquid nitrogen. Tissues were homogenized in ice-cold TRIS-EDTA buffer (PH 7.4) with protease inhibitors (Sigma-Aldrich, USA). Protein was estimated by Bradford method using Pierce BCA protein assay kit (Thermo Fisher scientific, USA).. Equal amounts of protein extract were denatured and separated on 4-12% NuPAGE Bis-Tris Gels and transferred on to PVDF membranes (Invitrogen, UK), which were then probed with rabbit anti-ZO-1 (ThermoFisher Scientific, USA) and rabbit anti-β-actin (abcam, USA) with HRP-conjugated secondary antibody. The bands were visualized using an enhanced ECL detection kit (Amersham, UK) and quantified by densitometry.

### Statistical analysis

Statistical analysis was performed using GraphPad Prism 6.0 (SanDiego, CA) and STATA version 11.0. Normality of data was tested using D’Agostino & Pearson omnibus normality test. Qualitative variables are presented as number and percentage while quantitative variables are presented as Mean ± SD and Median (Interquartile Range [IQR]) for normal and abnormal distribution respectively. For a comparison between cases and controls, two-tailed unpaired t-tests or a Mann–Whitney test were used wherever appropriate. For more than two groups one-way ANOVA or Kruskal-Wallis test was used. Spearman’s correlation was done to assess the correlation between different parameters. *P* < 0.05 was considered as statistical significance.

## Results

### Baseline clinical and tumor characteristics

Clinical and radiological characteristics of HCC patients enrolled in this study were presented in Table 1. Disease severity was characterized in HCC patients and was found to be distributed as Child-Pugh class A (30%), B (40%) and C (30%). According to BCLC staging, HCC patients were classified as stage A (0%), stage B (50%), stage C (20%) and stage D (30%). Among HCC patients, 57% of population had single lesion, 23% had two lesions, 6% had three lesions and 13% had multifocal lesions. 23% population had tumor size ≤5 cm and 77% of them had tumor size > 5 cm among total HCC patients enrolled. Metastasis was present in 27% cases and was absent in majority of the cases (73%). Among total HCC cases recruited, ~ 77% of them got cirrhosis and was absent in 23% cases. 50% of HCC cases had no hepatitis infection, 33% were HBs Ag positive and 17% were HCV positive. 27% of HCC patients had AFP level ≤ 15 (ng/ml) while 73% of population had > 15 (ng/ml).

**Table 1:** Clinical and radiological characteristics of HCC patients

Characters	N	%
Child-Pugh class		
A	9	30
B	12	40
C	9	30
BCLC staging		
Stage A	0	0
Stage B	15	50
Stage C	6	20
Stage D	9	30
Number of Lesions		
Single lesion	17	57
Two lesions	7	23
Three lesions	2	7
Multifocal	4	13
Tumor Size		
≤ 5 cm	7	23
> 5 cm	23	77
Metastasis		
Present	8	27
Absent	22	73
Cirrhosis		
Present	23	77
Absent	7	23
Hepatitis infection		
No hepatitis infection	15	50
HBs Ag positive	10	33
HCV positive	5	17
AFP level		
≤ 15 (ng/ml)	8	27
> 15 (ng/ml)	22	73
Total	30	100

*BCLC* Barcelona Clinic Liver Cancer, *AFP* alpha-fetoprotein

### General biochemical parameters in healthy volunteers and HCC patients

Age, gender and serum liver function test parameters in healthy volunteers and HCC patients were given in Table 2. The median age of controls were 55.0 (48.75-58.00) years and HCC patients were 56.50 (50.75-62.00) years, which were not found to be statistically significant (*P = 0.*213). The male and female ratio was 20/10 in both controls and HCC patients. The serum total and direct bilirubin concentrations were significantly (*p* < 0.0001 for both) higher in HCC patients compared to controls. Furthermore, the observed hepatic marker enzymes such as AST, ALT, γGT and ALP concentrations were significantly (*p* < 0.0001) increased in HCC patients whilst serum total protein and albumin concentrations were significantly decreased (*p* = 0.01 and *p* < 0.0001, respectively) when compared to healthy volunteers.

**Table 2:** Age, gender and general biochemical parameters in control subjects and HCC patients

Parameters	Controls (n = 30)	HCC patients (*n* = 30)	*Mann-Whitney Test*
			*p* value
Age (years)			
Mean ± SD	53.47 ± 6.64	54.37 ± 11.33	
Median (IQR)	55.0 (48.75-58.00)	56.50 (50.75-62.00)	0.213
Gender (Male/Female)	20/10	20/10	
Total bilirubin (mg/dl)			
Mean ± SD	0.71 ± 0.09	2.50 ± 0.4.65	
Median (IQR)	0.70 (0.68-0.73)	1.20 (0.78-1.73)	< 0.0001
Direct bilirubin (mg/dl)			
Mean ± SD	0.23 ± 0.05	0.77 ± 1.19	
Median (IQR)	0.20 (0.20-0.30)	0.40 (0.20-0.80)	< 0.0001
Total protein (gm/dl)			
Mean ± SD	7.33 ± 0.36	6.79 ± 0.94	< 0.01
Median (IQR)	7.30 (7.20-7.60)	6.85 (6.18-7.55)	
Albumin (gm/dl)			
Mean ± SD	3.97 ± 0.32	3.14 ± 0.67	
Median (IQR)	4.10 (3.60-4.30)	3.05 (2.68-3.60)	< 0.0001
AST (IU/L)			
Mean ± SD	22.90 ± 6.61	118.2 ± 99.87	
Median (IQR)	21.00 (19.75-28.00)	78.00 (51.00-148.5)	< 0.0001
ALT (IU/L)			
Mean ± SD	24.90 ± 8.40	60.79 ± 38.13	
Median (IQR)	23.00 (19.00-32.25)	45.00 (35.00-79.50)	< 0.0001
γGT (IU/L)			
Mean ± SD	27.47 ± 10.46	142.5 ± 136.0	
Median (IQR)	27.00 (19.50-32.00)	92.50 (57.00-159.0)	< 0.0001
ALP (IU/L)			
Mean ± SD	176.2 ± 30.55	663.0 ± 654.2	
Median (IQR)	180.0 (155.8-196.3)	480.5 (282.8-680.0)	< 0.0001

*ALT* alanine transaminase, *AST* aspartate transaminase, *γGT* gamma glutamyl transpeptidase, *ALP* alkaline phosphatase

### Correlation of ZO-1 with disease severity in HCC patients

Table 3 shows the correlation between plasma ZO-1 and HCC progression. There was a significant positive correlation between ZO-1 and child-Pugh class (*r* = 0.787 with *P* < 0.0001) in HCC patients. We also found significant positive correlation for ZO-1 with BCLC staging in HCC patients (*r* = 0.786 with P < 0.0001). Similarly, number of lesions and tumor size showing significant positive correlation between ZO-1 and HCC severity (*r* = 0.697, *P* < 0.0001; *r* = 0.561, *P* = 0.0029, respectively).

**Table 3:** Correlation of ZO-1 with disease severity in HCC patients

Parameter	*r*	*P* value
Child-Pugh class	0.7871	< 0.0001
BCLC stage	0.7864	< 0.0001
No. of lesions	0.6969	< 0.0001
Tumor size	0.5607	0.0029

r = coefficient of correlation. *BCLC* Barcelona clinic liver cancer, *HCC* hepatocellular carcinoma, *ZO-1* zonula occludens

### Correlation of hsCRP with disease severity in HCC patients

Table 4 shows the correlation between serum hsCRP and HCC severity. There were no significant positive correlation between hsCRP with child-Pugh class or BCLC staging, however, there were significant positive correlation between serum hsCRP and number of lesions and tumor size in HCC patients (*r* = 0.3989, *P* = 0.0435; *r* = 0.4469, *P* = 0.0221, respectively).

**Table 4:** Correlation of hs-CRP with disease severity in HCC patients

Parameter	*r*	*P* value
Child-Pugh class	0.2691	0.1838
BCLC stage	0.2687	0.1844
No. of lesions	0.3989	0.0435
Tumor size	0.4469	0.0221

*r* = coefficient of correlation, *BCLC* Barcelona clinic liver cancer, *HCC* hepatocellular carcinoma, *hs-CRP* high sensitivity C-reactive protein

### Blood ZO-1 concentration in healthy controls and HCC patients

**Fig. 1:**
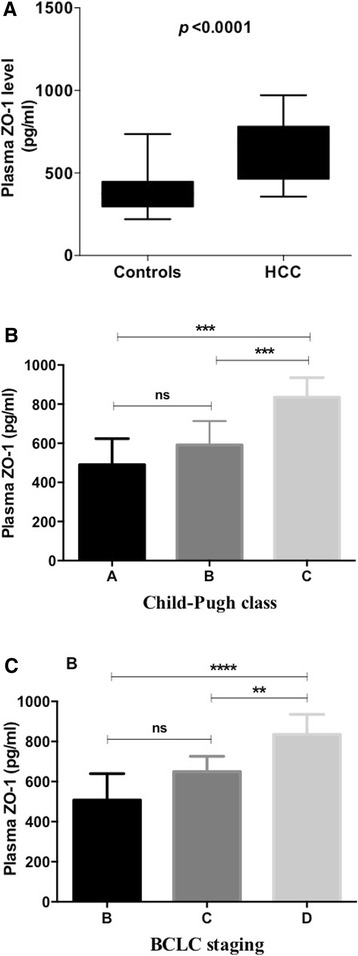
**a** Plasma ZO-1 levels in healthy volunteers and HCC patients. Values are expressed as mean ± standard deviation. *P* < 0.0001, controls Vs HCC. **b** Plasma ZO-1 levels according to Child-Pugh class in HCC patients. Values are expressed as mean ± standard deviation. ***P = 0.0003, Child-Pugh class A Vs C ***P = 0.0004, Child-Pugh class B Vs C; ns-No significant. **c** Plasma ZO-1 levels according to BCLC staging system in HCC patients. Values are expressed as mean ± standard deviation. ****P < 0.0001, BCLC staging B Vs D; **P < 0.01, BCLC staging C Vs D; ns-No significant

Compared to normal healthy volunteers, the plasma concentration of ZO-1 was significantly (*p* < 0.0001) elevated in HCC patients (Fig. 1a). Furthermore, we analysed increased ZO-1 concentration with HCC severity (Fig. 1b&c). According to Child-Pugh class, ZO-1 levels were increased with HCC progression [Child-Pugh class A (490.1 ± 133.5), Child-Pugh class B (591.5 ± 122.3) and Child-Pugh class C (835.3 ± 100.4)]. Plasma ZO-1 concentration was significantly increased in Child-Pugh class C patients when compared to both classes A&B patients (*P* < 0.001 for both), however no significant difference between Child-Pugh classes A&B were observed. We also analysed ZO-1 concentration based on the BCLC staging classification and found that significantly increased plasma ZO-1 levels in stage D HCC patients when compared to stages B and C patients (*P* < 0.0001 and *P* < 0.01, respectively). Furthermore, no statistical significant difference of ZO-1 levels found between stage B and stage C patients (Fig. 1c).

### Blood hsCRP concentration in healthy controls and HCC patients

**Fig. 2:**
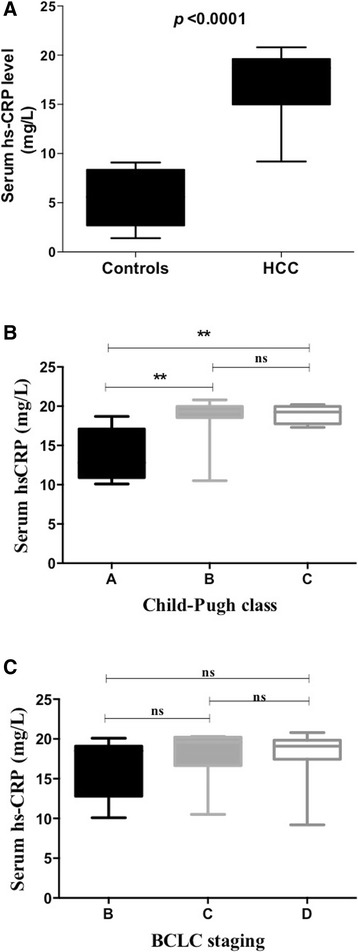
**a** Serum hs-CRP levels in healthy volunteers and HCC patients. Values are expressed as mean ± standard deviation. *p* < 0.0001, controls Vs HCC. **b** Serum hs-CRP levels according to Child-Pugh class in HCC patients. Values are expressed as mean ± standard deviation. **P < 0.01, Child-Pugh class A Vs B and A Vs C, respectively. **c** Serum hs-CRP levels according to BCLC staging system in HCC patients. Values are expressed as mean ± standard deviation. ns-No significant

Compared to healthy volunteers, the serum hsCRP levels were significantly (*p* < 0.0001) elevated in HCC patients (Fig. 2a). We also analysed increased serum hsCRP concentration with HCC severity (Fig. 2b&c). According to Child-Pugh classification, the observed hsCRP levels were increased with HCC progression (Fig. 2b). When compared to child-Pugh class A, hsCRP levels were significantly increased in child-Pugh classes B&C patients (*P* < 0.01 for both) however, no significant difference between Child-Pugh classes B&C were observed. Furthermore, hsCRP concentration was analysed with BCLC staging classification, and found no statistical significant difference among stages B, C and D patients (Fig. 2c).

### Correlation between increased ZO-1 and hsCRP concentrations in HCC patients

**Fig. 3:**
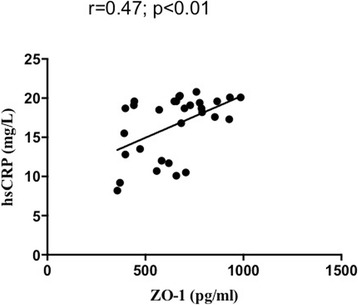
Prognostic potential of serum ZO-1 in HCC patients. Serum ZO-1 levels were positively correlated with increased hsCRP in HCC. Correlation coefficient (r) and statistical significance (*p* value) are indicated. Individual samples are represented as dots (*n* = 28)

The correlation between systemic ZO-1 and hsCRP in HCC patients was shown in Fig. 3. A statistically significant positive correlation was found between elevated levels of plasma ZO-1 and serum hsCRP (*r* = 0.47 and *p* < 0.01). However, no association was found between ZO-1 and other clinicopathological parameters.

### Receiver operator characteristics curve analysis of ZO-1 and hs-CRP in HCC patients

**Fig. 4:**
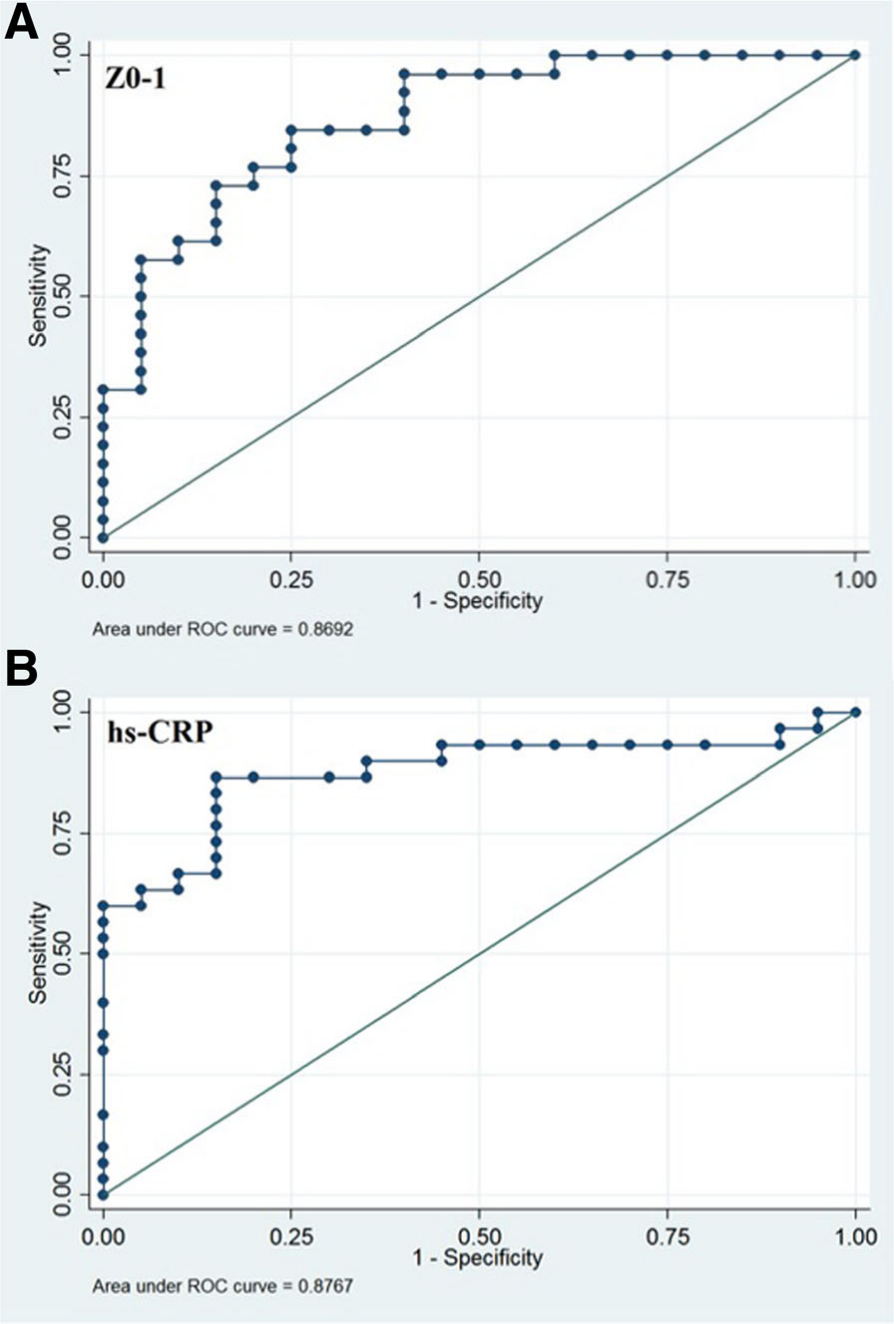
ROC curve analysis of ZO-1 (**a**) and hs-CRP (**b**). ZO-1 at cut off of 472.99 (pg/ml) had a sensitivity of 76.92%, specificity of 80.00% with AUC of 0.8692. hs-CRP at cut off of 9.2 (mg/L) had a sensitivity of 86.67%, specificity of 85.00% with Area Under Curve of 0.8767

Table 5 and Fig. 4 show the ROC curve analysis of both ZO-1 and hsCRP in HCC patients. ZO-1 at cut off of 472.99 pg/ml had 76.92% sensitivity and 80% specificity. and area under curve (0.8692) with 95% confidence interval (0.766-0.972), positive likelihood ratio of 3.84 and negative likelihood ratio of 0.28 for the population under study. Similarly, hs-CRP at cut off of 9.2 mg/L had 86.67% sensitivity, 85.0% specificity and area under curve (0.8767) with 95% confidence interval (0.776 – 0.976), positive likelihood ratio (5.77) and negative likelihood ratio (0.15) for the population under study.

**Table 5:** ROC curve analysis of ZO-1 and hs-CRP

Parameters	Cut off value	Sensitivity (%)	Specificity (%)	AUC	95% CI	LR+	LR-
ZO-1 (pg/ml)	472.99	76.92	80.00	0.8692	0.766 – 0.972	3.84	0.28
hs-CRP (mg/L)	9.2	86.67	85.00	0.8767	0.776 - 0.976	5.77	0.15

*AUC* area under curve, *CI* confidence interval, *hsCRP* high sensitivity C-reactive protein, *LR+* positive likelihood ratio, *LR-* negative likelihood ratio, *ROC* receiver operator characteristics

### Expression of ZO-1 in HCC liver and noncancerous liver specimen obtained from HCC patients

**Fig. 5:**
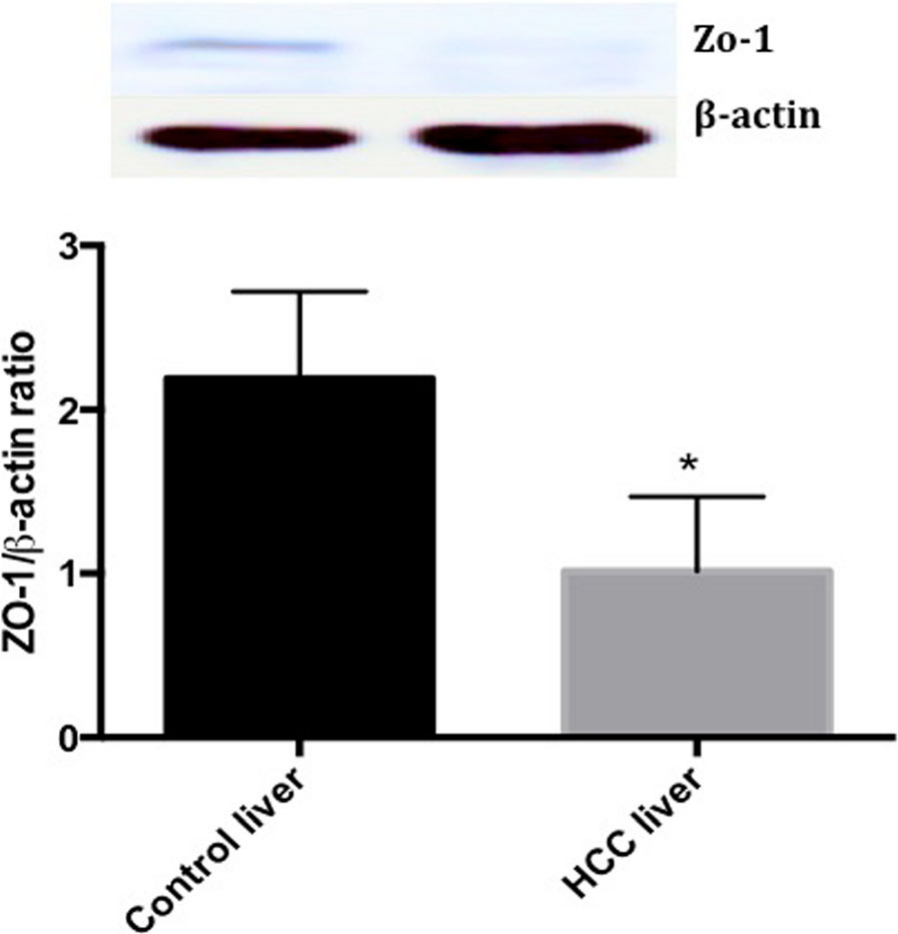
Protein expression of ZO-1 in HCC liver and noncancerous liver tissues (histologically proven control) from HCC patients. Values are expressed as mean ± standard deviation. *P* < 0.05, controls Vs HCC

**Fig. 6:**
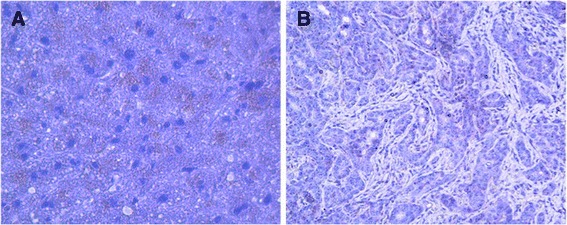
Immunohistochemical localization of ZO-1 in noncancerous liver tissue (histologically proven control) (**a**) and HCC liver (**b**) from HCC patients. ZO-1 is not expressed in HCC liver (**b**), whereas control liver tissue (**a**) expressed increased ZO-1 particularly in the bile ducts

Hepatic expression of ZO-1 was analysed by western blotting (Fig. 5). ZO-1 expression was increased significantly (*p* < 0.05) in noncancerous liver specimen (histologically proven control) obtained from HCC patients compared to HCC liver obtained form the same patients. Moreover, immunohistochemical study show that ZO-1 cellular expression is completely absent in HCC liver (Fig. 6a) whereas noncancerous liver specimen (histologically proven control) obtained from HCC patients showed a positive spot and strongly appeared on bile canaliculi (Fig. 6b).

## Discussion

The results of this study demonstrated for the first time in HCC patients that increased blood levels of ZO-1 was correlated significantly with increased hsCRP concentrations observed in the same patient samples. Since ZO-1 is a first tight junction protein identified and its expression was shown to decrease in many cancers, including HCC [20]. In this regard, a very recent report indicates in HCC patients after hepatic resection that the low expression of ZO-1 was significantly associated with poor survival [21]. We found similar observation in HCC patient liver, which shows very faint ZO-1 expression compared to noncancerous liver tissue collected from the same HCC patients. Increased blood ZO-1 concentration observed in HCC further supports the hypothesis that decreased hepatic-intestinal barrier dysfunction with marked ZO-1 and other TJ protein disruptions. This finding is also consistent with a very recent study showing in ischemic reperfusion (I/R) injury model that low ZO-1 expression contributing to TJ disruption and increased gut permeability [22] following intestinal I/R insult. Further, it has been reported in response to proinflammatory cytokines, the expression of ZO-1 was reduced and redistributed away from the TJ upon increased intestinal permeability [22, 23]. TNF α a known proinflammatory cytokine was shown to inhibit the expression of ZO-1 through the mechanism involved in the triggering nuclear factor-κ B (NF-κB) thus, increased intestinal epithelial TJ permeability [22, 24]. Moreover, decreased intestinal TJ integrity, a key pathogenic feature involved in the progression of intestinal inflammation [13, 23]. Thus, we speculate the loss of TJ protein during HCC progression on the background of inflammation [20, 24], consequently released in the circulation, perhaps increased plasma ZO-1 in observed in HCC patients.

A growing body of literature showing evidence that a solid pathological association between chronic low-grade inflammation and carcinogenesis [25]. High sensitive CRP, an acute-phase reactant synthesized in the liver that is regulated by pro-inflammatory cytokines, primarily interleukin (IL) 6 [26]. It has been reported to be associated with a poor prognosis in patients with different types of cancer, which including HCC [26]. In this study, hsCRP was found to elevated in HCC patients when compared to healthy volunteers. In this context, the previous studies showing compelling evidence that systemic inflammation is known to associate with tumor development and poor survival in patients with HCC [3, 11]. In addition, a moderate increase of hsCRP showed to predict recurrence and survival in patients with early-stage HCC [27]. Further, it has been demonstrated that a novel prognostic scoring system, which includes the CRP level, predict overall survival in late-stage HCC following treatment with Sorafenib [28]. Shin et al., found that increased serum CRP concentration indicates poor prognosis in patients with HCC [29]. Also, increased immunoreactivity of CRP is considered a key feature of inflammatory hepatic adenomas with an increased risk of malignant transformation [29]. Furthermore, the ratio of hsCRP and albumin predicted outcomes in patients with HCC [30] and considered a novel inflammation-based prognostic score. We found decreased albumin concentrations in HCC patients compared to healthy control. However, there is no correlation between elevated hsCRP and lowered albumin levels in HCC.

Alpha-fetoprotein (AFP), a plasma glycoprotein synthesized during early fetal life by the liver and considered HCC specific marker [31]. However, in the current study we found only 73% of HCC patients had elevated (> 15 ng/mL) serum AFP level whilst 27% had normal (< 15 ng/mL) AFP level consistent with previous report showing that AFP was not an optimal marker for the early detection of HCC patients on the background of hepatitis C infection [32]. This may indicate lower diagnostic efficacy of AFP in HCC however; it needs to prove with larger sample size. Hepatic function markers such as total bilirubin, ALT, AST, ALP, and GGT are widely used in clinical diagnosis of hepatic dysfunction and damage [33]. Increased concentration found in many acute and chronic liver ailments and also associated with HCC progression [34]. In the current study, the plasma concentration of ALT, AST, ALP, GGT and total and direct bilirubin were significantly (*p* < 0.0001) elevated in HCC patients when compared to control subjects. Indeed, none of the parameters correlated with increased blood ZO-1 concentration. A previous study has shown evidence that AST, ALT, and GGT were elevated in 90% of diagnosed HCC patients whilst half of the patients also showed increased bilirubin or liver-specific ALP concentrations, indicating these are good pre-diagnostic markers of liver cancer [35].

## Conclusion

The current study identified elevated serum ZO-1 concentration in HCC patients for the first time, and this was positively correlated with increased hsCRP levels. Thus, our study showing evidence that inflammation promotes plasma ZO-1 concentration and raises the possibility that it could be used as a potential diagnostic biomarker for HCC progression. Moreover, diminished hepatic expression of ZO-1 found in HCC patients further confirms that targeting ZO-1 possibly provides a rational approach to the management of HCC and may serve as a marker for future molecular phenotyping of HCC.

## Additional file

Additional file 1: **Figure S1.** Histopathology and cytology findings of liver specimen obtained from the HCC patient. Histopathology sections of Hepatocellular carcinoma a) arranged in trabeculae (H&Ex100); b) and pseudoglands (H&Ex100); c) on high power cells show moderate degree of nuclear atypia and mitotic figure (H&Ex400); d) IHC with Heppar1 shows strong cytoplasmic positivity (IHCx400). Cytology of same case shows e) highly cellular smears arranged in fragments, clusters and dispersed cells (Papx100); f) malignant cells traversed by capillary blood vessels (MGGx200). (JPG 132 kb)

## Abbreviations

AFP: Alpha-fetoprotein
ALP: Alkaline phosphatase
ALT: Alanine aminotransferase
AST: Aspartate aminotransferase
BCLC: Barcelona clinic liver cancer
HBs Ag: Hepatitis B surface antigen
HCC: Hepatocellular carcinoma
HCC: Hepatocellular carcinoma
HCV: Hepatitis C virus
hs-CRP: High sensitivity c-reactive protein
IQR: Inter-quartile range
SD: Standard deviation
ZO-1: Zonula occluden-1
γGT: Gamma glutamyl transaminase

## References

[1] El-Serag HB (2011). Hepatocellular carcinoma. N Engl J Med.

[2] Ghouri YA, Mian I, Rowe JH (2017). Review of hepatocellular carcinoma: epidemiology, etiology, and carcinogenesis. J Carcinog.

[3] Forner A, Llovet JM, Bruix J (2012). Hepatocellular carcinoma. Lancet.

[4] Ohnishi K, Iida S, Iwama S, Goto N, Nomura F, Takashi M, Mishima A, Kono K, Kimura K, Musha H (1982). The effect of chronic habitual alcohol intake on the development of liver cirrhosis and hepatocellular carcinoma: relation to hepatitis B surface antigen carriage. Cancer.

[5] Davila JA, Morgan RO, Shaib Y, McGlynn KA, El-Serag HB (2004). Hepatitis C infection and the increasing incidence of hepatocellular carcinoma: a population-based study. Gastroenterology.

[6] Barashi N, Weiss ID, Wald O, Wald H, Beider K, Abraham M, Klein S, Goldenberg D, Axelrod J, Pikarsky E, et al. Inflammation induced hepatocellular carcinoma is dependent on CCR5. Hepatology. 2013;58(3):1021-30. 10.1002/hep.26403 23526353

[7] El-Serag HB, Tran T, Everhart JE (2004). Diabetes increases the risk of chronic liver disease and hepatocellular carcinoma. Gastroenterology.

[8] Yu MC, Yuan JM (2004). Environmental factors and risk for hepatocellular carcinoma. Gastroenterology.

[9] Fazio C, Ricciardiello L (2016). Inflammation and notch signaling: a crosstalk with opposite effects on tumorigenesis. Cell Death Dis.

[10] Grinberg-Bleyer Y, Ghosh S (2016). A novel link between inflammation and Cancer. Cancer Cell.

[11] Bishayee A (2014). The role of inflammation and liver cancer. Adv Exp Med Biol.

[12] Uehara T, Ainslie GR, Kutanzi K, Pogribny IP, Muskhelishvili L, Izawa T, Yamate J, Kosyk O, Shymonyak S, Bradford BU (2013). Molecular mechanisms of fibrosis-associated promotion of liver carcinogenesis. Toxicol Sci.

[13] Zihni C, Mills C, Matter K, Balda MS (2016). Tight junctions: from simple barriers to multifunctional molecular gates. Nat Rev Mol Cell Biol.

[14] Sawada N, Murata M, Kikuchi K, Osanai M, Tobioka H, Kojima T, Chiba H (2003). Tight junctions and human diseases. Med Electron Microsc.

[15] Fanning AS, Anderson JM (1999). PDZ domains: fundamental building blocks in the organization of protein complexes at the plasma membrane. J Clin Invest.

[16] Itoh M, Furuse M, Morita K, Kubota K, Saitou M, Tsukita S (1999). Direct binding of three tight junction-associated MAGUKs, ZO-1, ZO-2, and ZO-3, with the COOH termini of claudins. J Cell Biol.

[17] Hoover KB, Liao SY, Bryant PJ (1998). Loss of the tight junction MAGUK ZO-1 in breast cancer: relationship to glandular differentiation and loss of heterozygosity. Am J Pathol.

[18] Kaihara T, Kusaka T, Nishi M, Kawamata H, Imura J, Kitajima K, Itoh-Minami R, Aoyama N, Kasuga M, Oda Y (2003). Dedifferentiation and decreased expression of adhesion molecules, E-cadherin and ZO-1, in colorectal cancer are closely related to liver metastasis. J Exp Clin Cancer Res.

[19] Kimura Y, Shiozaki H, Hirao M, Maeno Y, Doki Y, Inoue M, Monden T, Ando-Akatsuka Y, Furuse M, Tsukita S (1997). Expression of occludin, tight-junction-associated protein, in human digestive tract. Am J Pathol.

[20] Orban E, Szabo E, Lotz G, Kupcsulik P, Paska C, Schaff Z, Kiss A (2008). Different expression of occludin and ZO-1 in primary and metastatic liver tumors. Pathol Oncol Res.

[21] Nagai T, Arao T, Nishio K, Matsumoto K, Hagiwara S, Sakurai T, Minami Y, Ida H, Ueshima K, Nishida N (2016). Impact of tight junction protein ZO-1 and TWIST expression on postoperative survival of patients with hepatocellular carcinoma. Dig Dis.

[22] Shen ZY, Zhang J, Song HL, Zheng WP (2013). Bone-marrow mesenchymal stem cells reduce rat intestinal ischemia-reperfusion injury, ZO-1 downregulation and tight junction disruption via a TNF-alpha-regulated mechanism. World J Gastroenterol.

[23] Tian S, Guo R, Wei S, Kong Y, Wei X, Wang W, Shi X, Jiang H (2016). Curcumin protects against the intestinal ischemia-reperfusion injury: involvement of the tight junction protein ZO-1 and TNF-alpha related mechanism. Korean J Physiol Pharmacol.

[24] Ma TY, Iwamoto GK, Hoa NT, Akotia V, Pedram A, Boivin MA, Said HM (2004). TNF-alpha-induced increase in intestinal epithelial tight junction permeability requires NF-kappa B activation. Am J Physiol Gastrointest Liver Physiol.

[25] Lee CH, Chang JS, Syu SH, Wong TS, Chan JY, Tang YC, Yang ZP, Yang WC, Chen CT, Lu SC (2015). IL-1beta promotes malignant transformation and tumor aggressiveness in oral cancer. J Cell Physiol.

[26] Kinoshita A, Onoda H, Imai N, Nishino H, Tajiri H (2015). C-reactive protein as a prognostic marker in patients with hepatocellular carcinoma. Hepato-Gastroenterology.

[27] Fujiwara N, Tateishi R, Nakagawa H, Nakagomi R, Kondo M, Minami T, Sato M, Uchino K, Enooku K, Kondo Y (2015). Slight elevation of high-sensitivity C-reactive protein to predict recurrence and survival in patients with early stage hepatitis C-related hepatocellular carcinoma. Hepatol Res.

[28] Nakanishi H, Kurosaki M, Tsuchiya K, Yasui Y, Higuchi M, Yoshida T, Komiyama Y, Takaura K, Hayashi T, Kuwabara K (2016). Novel pretreatment scoring incorporating C-reactive protein to predict overall survival in advanced hepatocellular carcinoma with Sorafenib treatment. Liver Cancer.

[29] Shin JH, Kim CJ, Jeon EJ, Sung CO, Shin HJ, Choi J, Yu E (2015). Overexpression of C-reactive protein as a poor prognostic marker of Resectable hepatocellular carcinomas. J Pathol Transl Med.

[30] Kinoshita A, Onoda H, Imai N, Iwaku A, Oishi M, Tanaka K, Fushiya N, Koike K, Nishino H, Matsushima M (2015). The C-reactive protein/albumin ratio, a novel inflammation-based prognostic score, predicts outcomes in patients with hepatocellular carcinoma. Ann Surg Oncol.

[31] Spangenberg HC, Thimme R, Blum HE (2006). Serum markers of hepatocellular carcinoma. Semin Liver Dis.

[32] Lok AS, Sterling RK, Everhart JE, Wright EC, Hoefs JC, Di Bisceglie AM, Morgan TR, Kim HY, Lee WM, Bonkovsky HL (2010). Des-gamma-carboxy prothrombin and alpha-fetoprotein as biomarkers for the early detection of hepatocellular carcinoma. Gastroenterology.

[33] Stepien M, Fedirko V, Duarte-Salles T, Ferrari P, Freisling H, Trepo E, Trichopoulou A, Bamia C, Weiderpass E, Olsen A (2016). Prospective association of liver function biomarkers with development of hepatobiliary cancers. Cancer Epidemiol.

[34] Schlesinger S, Aleksandrova K, Pischon T, Jenab M, Fedirko V, Trepo E, Overvad K, Roswall N, Tjonneland A, Boutron-Ruault MC (2013). Diabetes mellitus, insulin treatment, diabetes duration, and risk of biliary tract cancer and hepatocellular carcinoma in a European cohort. Ann Oncol.

[35] Lopez JB, Balasegaram M, Thambyrajah V, Timor J (1996). The value of liver function tests in hepatocellular carcinoma. Malays J Pathol.

