# Supplementary figures and images for: Increased systemic zonula occludens 1 associated with inflammation and independent biomarker in patients with hepatocellular carcinoma
Source: BMC Cancer. 2018 May 18;18:572. doi: 10.1186/s12885-018-4484-5 (PMC5960107; doi:10.1186/s12885-018-4484-5)

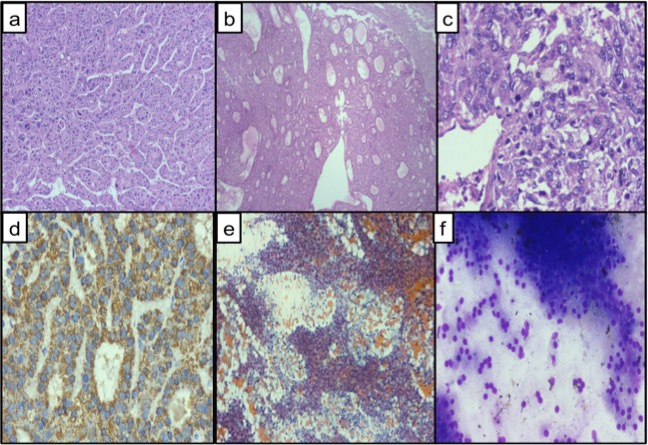

Supplement: Supplementary file 1 — Figure S1. Histopathology and cytology findings of liver specimen obtained from the HCC patient. Histopathology sections of Hepatocellular carcinoma a) arranged in trabeculae (H&Ex100); b) and pseudoglands (H&Ex100); c) on high power cells show moderate degree of nuclear atypia and mitotic figure (H&Ex400); d) IHC with Heppar1 shows strong cytoplasmic positivity (IHCx400). Cytology of same case shows e) highly cellular smears arranged in fragments, clusters and dispersed cells (Papx100); f) malignant cells traversed by capillary blood vessels (MGGx200). (JPG 132 kb) [file 12885_2018_4484_MOESM1_ESM.jpg]
